# Comparison of Outcomes Between Sacubitril/Valsartan and Enalapril in Patients With Heart Failure: A Systematic Review and Meta-Analysis

**DOI:** 10.7759/cureus.48623

**Published:** 2023-11-10

**Authors:** Ibrahim Reyaz, Avneet Kaur, Moyal Z Saad, Manisha Kanumuri, Hari Priya Nistala, Sumyyia Usman, Celene Olivas, Sujith K Palleti

**Affiliations:** 1 Internal Medicine, Christian Medical College and Hospital Ludhiana, Punjab, Ludhiana, IND; 2 Internal Medicine, Government Medical College Patiala, Patiala, IND; 3 Internal Medicine, Jinnah Medical and Dental College, Karachi, PAK; 4 Department of Psychiatry, Mediciti Institute of Medical Sciences, Hyderabad, IND; 5 Internal Medicine, Maharajah’s Institute of Medical Sciences, Vizianagaram, IND; 6 Medicine, Dow University of Health Sciences, Karachi, PAK; 7 Medicine, National Autonomous University of Nicaragua, Mexico City, MEX; 8 Nephrology, Louisiana State University Health Sciences Center, Shreveport, USA

**Keywords:** systematic review and meta analysis, cardiovascular outcomes, heart failure, sacubitril–valsartan, enalapril

## Abstract

The objective of this meta-analysis was to compare outcomes between sacubitril/valsartan and enalapril in patients with heart failure. We performed this meta-analysis according to the guidelines reported in the Preferred Reporting Items for Systematic Reviews and Meta-Analyses (PRISMA) statement. Two independent authors systematically searched online databases including PubMed, Cochrane Library, and Web of Science from inception till September 15, 2023. Outcomes assessed in this meta-analysis included all-cause mortality, cardiovascular mortality, and cardiovascular-related hospitalization. A total of nine studies were included in this meta-analysis. Pooled analysis showed that the risk of all-cause mortality was higher in patients receiving enalapril compared to patients receiving sacubitril/valsartan (risk ratio [RR]: 0.57; 95% CI: 0.31 to 1.04). Risk of cardiovascular mortality was significantly higher in the enalapril group compared to the sacubitril/valsartan group (RR: 0.75; 95% CI: 0.62 to 0.91). The risk of cardiovascular hospitalization was significantly higher in the enalapril group compared to the sacubitril/valsartan group (RR: 0.76; 95% CI: 0.66 to 0.86). In conclusion, our meta-analysis of nine studies underscores the superior clinical performance of sacubitril/valsartan compared to enalapril in managing patients with heart failure.

## Introduction and background

Heart failure (HF), characterized by any structural or functional impairment of ventricular filling or ejection of blood, is a leading global cause of morbidity and mortality [1]. In the United States, HF affects more than 5.1 million individuals, with 650,000 new HF cases annually [1]. HF patients face a higher risk of mortality and hospitalization compared to patients with other heart conditions [1]. The prevalence of HF rises after the age of 60, particularly in individuals with coronary artery disease, hypertension, and other cardiac comorbidities [2]. Common HF symptoms include dyspnea, orthopnea, fatigue, reduced exercise tolerance, ankle swelling, and other signs of fluid overload [3].

Sacubitril/valsartan, an innovative angiotensin receptor-neprilysin inhibitor, has been approved for the treatment of heart failure with reduced ejection fraction (HFrEF) due to its demonstrated superiority over the angiotensin-converting enzyme inhibitor (ACEi) enalapril in the pivotal PARADIGM-HF trial [4]. In this trial, sacubitril/valsartan reduced all-cause mortality by 16% and decreased the rate of HF-related hospitalizations by 21% compared to standard ACEi therapy in symptomatic HFrEF patients [5]. Additionally, sacubitril/valsartan showed significant improvements in echocardiography results, vital signs, and biomarkers in chronic HF patients. It also led to a reduction in hyperkalemia, renal problems, and other adverse effects compared to traditional medications, resulting in fewer hospitalizations [6].

Furthermore, a meta-analysis conducted in 2021, which included five relevant randomized controlled trials (RCTs), concluded that sacubitril/valsartan improved left ventricular ejection fraction in HF patients, as indicated by a standardized mean deviation of 1.1 (95% CI = 1.01 to 1.19; p < 0.00001; fixed-effects model). Additionally, it significantly reduced the left ventricular volume index and lowered the risk of cardiovascular death (RR = 0.89; 95% CI = 0.83 to 0.96; p = 0.003) and HF-related rehospitalization (RR = 0.83; 95% CI = 0.78 to 0.88; p < 0.01) when compared to the control group [7].

Enalapril falls in the category of angiotensin-converting enzyme (ACE) inhibitors. Its mode of action revolves around the inhibition of ACE, an enzyme responsible for constricting blood vessels and elevating blood pressure [8]. Enalapril's role is to obstruct ACE, which, in turn, fosters vasodilation, leading to lowered blood pressure and decreased strain on the heart. Consequently, it proves valuable in the treatment of heart conditions such as hypertension and HF [9]. Previous research has demonstrated enalapril's significant impact on the exercise capacity of patients with HFrEF [10-11]. In comparison to a placebo, enalapril enhances peak oxygen consumption (VO2) and exercise duration after a 12-week treatment period [10]. A recent study by Piepoli et al. found no substantial advantage of sacubitril/valsartan over enalapril (10 mg bid) in terms of the 6-minute walk test (6-MWT) or daytime physical activity after 12 weeks [12].

Surprisingly, there has been a lack of comprehensive reviews addressing various aspects of efficacy and safety parameters on this subject to date. Additionally, existing primary studies have provided somewhat inconclusive evidence. Therefore, we conducted an exhaustive systematic review and meta-analysis to explore the roles of sacubitril/valsartan and enalapril in managing patients with HF. The objective of this meta-analysis was to make a comparison between sacubitril/valsartan and enalapril in patients with HF.

## Review

Methodology

We performed this meta-analysis according to the guidelines reported in the Preferred Reporting Items for Systematic Reviews and Meta-Analyses (PRISMA) statement.

Literature Search Strategy

Two independent authors systematically searched online databases including PubMed, Cochrane Library, and Web of Science from inception till September 15, 2023. The keywords used to search for relevant studies included the following: “Enalapril,” “Sacubitril/valsartan,” and “heart failure.” To carry out the search comprehensively, we merged medical subject headings (MeSH) and free-text headings. We used the appropriate Boolean operators including “AND,” “OR,” “NOT.” Additionally, reference lists of the included studies were manually searched to find additional studies relevant to the study topic. Other data sources were also searched, such as databases of grey/unpublished/unprinted literature, relevant reviews, and editorials.

Study Design and Selection Criteria

The eligibility and article inclusion/exclusion process followed a hierarchical approach, involving a thorough evaluation of the title, abstract, and full text. The selection and critical appraisal of studies adhered to the Joanna Briggs Institute's (JBI) protocol, which offers a more precise and stringent set of criteria for the study selection process. The pre-defined inclusion criteria were observational studies (retrospective cohorts and prospective cohorts) or RCTs comparing sacubitril/valsartan and enalapril in patients with HF and reported one of the required outcomes. The pre-defined exclusion criteria included (1) studies that involved animals, (2) reports, case series, review articles, and editorials, and (3) studies without a control or comparison group.

Data Extraction

All included articles from the systematic search were imported into EndNote X9 Reference Manager (Clarivate Analytics, Philadelphia, PA), where duplicates from various online databases were removed. Subsequently, two independent researchers conducted a thorough assessment of the remaining articles, selecting only those that met the predetermined inclusion criteria. Initially, titles and abstracts of all studies were screened, followed by a full-text review to assess relevance. Any discrepancies were resolved through discussion with a third researcher. Data were collected on study characteristics (e.g., author, publication year, region/hospital, study design, follow-up period) and population characteristics (e.g., sample size, mean age, gender distribution, baseline comorbidities such as diabetes and hypertension) for two study groups. Outcomes assessed in this meta-analysis included all-cause mortality, cardiovascular mortality, and cardiovascular-related hospitalization.

Statistical Analysis

We conducted all statistical analyses using Review Manager (version 5.4; Copenhagen: The Nordic Cochrane Centre, The Cochrane Collaboration, 2022). The outcomes were expressed as risk ratios (RRs) along with their corresponding 95% confidence intervals and were combined utilizing a random-effects model based on inverse variance weighting. To assess heterogeneity, we employed both the chi-square test of heterogeneity and the I-square statistic. A p-value of less than 0.1 indicated significant heterogeneity, while the I-square value quantified the extent of heterogeneity.

Results

**Figure 1:**
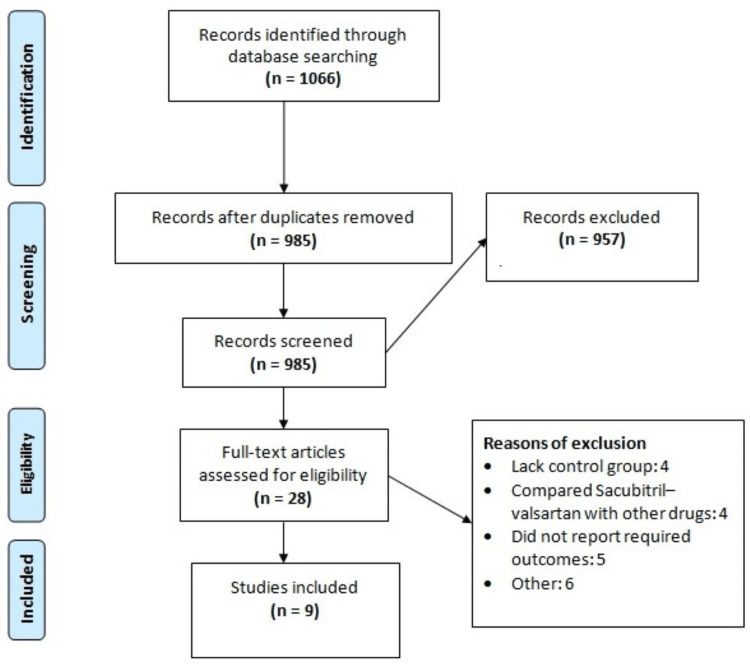
PRISMA flowchart of study selection PRISMA, Preferred Reporting Items for Systematic Reviews and Meta-Analyses

Figure 1 shows the process of study selection. We obtained 1,066 studies through online database searching. After removing duplicates, 985 studies were initially screened using abstracts or titles. Full texts of 28 studies were thoroughly screened using pre-defined inclusion and exclusion criteria. Finally, nine studies were included in this meta-analysis. Out of nine studies, eight were RCTs. Table 1 shows the characteristics of the included studies.

**Table 1:** Characteristics of the included studies RCT, randomized controlled trial; RC, retrospective cohort; NR, not reported

Author name	Year	Design	Groups	Dose	Sample Size	Follow-up	Age in years (Mean)	Males (n)	Hypertension (n)	Diabetes (n)
Bano et al. [13]	2021	RCT	Sacubitril–valsartan	50 or 100 mg twice	181	12 months	53	88	165	68
			Enalapril	2.5 or 5 mg twice	183		55	90	170	65
Desai et al. [14]	2019	RCT	Sacubitril–valsartan	200 mg twice	103	3 months	67.8	170	NR	NR
			Enalapril	10 mg twice	98		66.7	185		
Li et al. [15]	2019	RCT	Sacubitril–valsartan	NR	62	6 months	NR	NR	NR	NR
			Enalapril	NR	64					
McMurray et al. [4]	2014	RCT	Sacubitril–valsartan	200 mg twice	4187	27 months	63.8	3,308	2,969	1451
			Enalapril	10 mg twice	4212		63.8	3,259	2,971	1456
Pathadka et al. [16]	2020	RC	Sacubitril–valsartan	NR	1056	5.3 months	65.8	749	384	265
			Enalapril	NR	2181		78.6	1,114	1,163	654
Piepoli et al. [12]	2021	RCT	Sacubitril–valsartan	200 mg twice	309	4 months	67.2	238	213	96
			Enalapril	10 mg twice	310		66.6	249	203	117
Tsutsui et al. [17]	2021	RCT	Sacubitril–valsartan	200 mg twice	111	33.9 months	69	96	71	52
			Enalapril	10 mg twice	112		66.7	96	82	52
Velazquez et al. [18]	2019	RCT	Sacubitril–valsartan	200 mg twice	440	2 months	61	327	329	NR
			Enalapril	10 mg twice	441		63	308	309	NR
Zhao et al. [19]	2022	RCT	Sacubitril–valsartan	50 mg twice	52	6 months	68.6	23	32	34
			Enalapril	10 mg once	45		66.7	20	27	25

All-Cause Mortality

**Figure 2:**
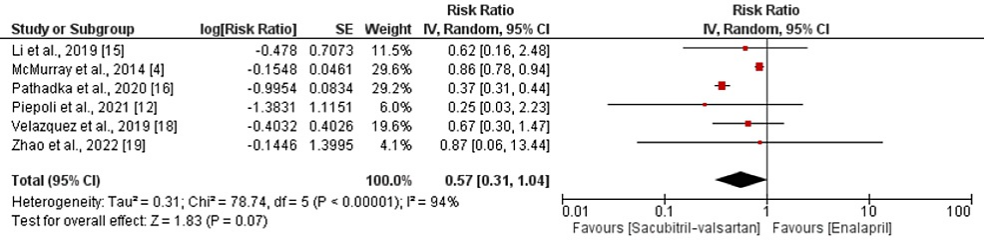
Comparison of all-cause mortality between the two groups Source: [4,12,15-16,18-19]

Six studies were included in the pooled analysis of all-cause mortality. The risk of all-cause mortality was higher in patients receiving enalapril compared to patients receiving sacubitril/valsartan (RR: 0.57; 95% CI: 0.31 to 1.04), as shown in Figure 2. Significant heterogeneity was reported among the study results (p = 0.001; I-square: 94%). We performed sensitivity analysis by removing the study conducted by Pathadka et al. as it was a retrospective study. After removing this study, the risk was still higher in the enalapril group; however, the difference between the two groups was statistically significant (RR: 0.85; 95% CI: 0.78 to 0.93). Heterogeneity among the study results reduced from 94% to 0%.

Cardiovascular Mortality

**Figure 3:**
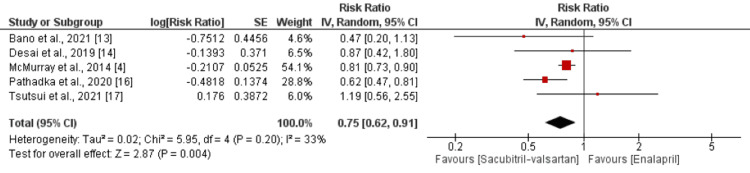
Comparison of cardiovascular mortality between the two groups Source: [4,13-14,16-17]

Five studies were included in the pooled analysis of comparison of cardiovascular mortality in the sacubitril/valsartan and enalapril groups. As shown in Figure 3, the risk of cardiovascular mortality was significantly higher in the enalapril group compared to the sacubitril/valsartan group (RR: 0.75; 95% CI: 0.62 to 0.91). No significant heterogeneity was reported among the study results (I-square: 33%; p = 0.20).

Cardiovascular Hospitalization

**Figure 4:**
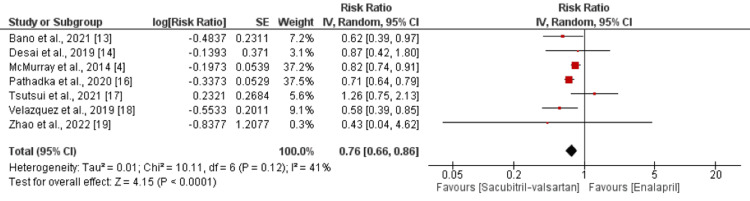
Comparison of cardiovascular hospitalization between the two groups Source: [4,13-14,16-19]

Seven studies compared the risk of cardiovascular hospitalization between the sacubitril/valsartan and enalapril groups. As shown in Figure 4, the risk of cardiovascular hospitalization was significantly higher in the enalapril group compared to the sacubitril/valsartan group (RR: 0.76; 95% CI: 0.66 to 0.86). No significant heterogeneity was reported among the study results (p = 0.12; I-square: 41%).

Discussion

Extensive research has explored the use of sacubitril/valsartan for treating patients with HF, leading to recommendations for its inclusion as a crucial component in managing such patients. Consequently, when considering both sacubitril/valsartan and enalapril, both medications are used for HF treatment; however, they operate through distinct mechanisms and offer varying potential advantages. Comparative studies serve to elucidate these differences, shedding light on their respective strengths and weaknesses, ultimately enhancing patient care and informing treatment protocols.

A total of nine studies were included in this meta-analysis, with eight of them being RCTs. The present meta-analysis reported that the risk of all-cause mortality, cardiovascular mortality, and cardiovascular hospitalization was significantly higher in patients receiving enalapril compared to patients receiving sacubitril/valsartan. This finding aligns with previous reviews comparing the efficacy of sacubitril/valsartan with other drugs regarding hospitalization and mortality outcomes in patients with HF [7,20]. The number of clinical trials comparing the efficacy of sacubitril/valsartan and enalapril in patients with HF is limited; therefore, more trials are needed to better understand the efficacy of these drugs in patients with HF.

The PARADIGM-HF trial [4] is one of the largest clinical trials comparing sacubitril/valsartan and enalapril. It reported a decreased risk of the composite outcome of HF hospitalization and cardiovascular mortality in the sacubitril/valsartan group by 20% compared to the enalapril group. Hospitalization is a strong predictor of quality of life and survival in patients with HF [21]. Past studies have also shown the cost-effectiveness of sacubitril/valsartan compared to ACE inhibitors and angiotensin II receptor Blockers (ARBs), reflecting lower healthcare costs associated with sacubitril/valsartan [22-23]. Thus, sacubitril/valsartan could enhance outcomes in patients with HF and optimize the utilization of healthcare resources.

There is limited research on the use of sacubitril/valsartan in older individuals. The risk of mortality is higher in individuals with HF aged more than 65 years compared to their younger counterparts [24]. In a retrospective study conducted by Pathadka et al. [16], the mean age of patients was almost 12 years higher compared to the PARADIGM-HF trial population, with more than 75% of the population being 65 years old or older. This study supported the evidence that sacubitril/valsartan is more effective in terms of reducing the rate of hospitalization and all-cause mortality. However, due to the lack of individual-level data, we were unable to perform subgroup analysis. Additional research should be conducted to investigate the safety of sacubitril/valsartan in elderly individuals and those with kidney problems, as well as any potential obstacles to the widespread clinical use of this medication.

One of the limitations of this meta-analysis is that we were not able to conduct a subgroup analysis due to a lack of individual-level data. Secondly, the follow-up of majority of the studies was less than six months. Therefore, in the future, more prospective trials are required to compare the long-term outcomes between the sacubitril/valsartan and enalapril groups.

## Conclusions

In conclusion, our meta-analysis of nine studies underscores the superior clinical performance of sacubitril/valsartan compared to enalapril in managing patients with HF. Notably, sacubitril/valsartan demonstrated a significant advantage over enalapril in all-cause mortality, cardiovascular mortality, and cardiovascular hospitalization. These findings, supported by previous reviews, highlight the potential for sacubitril/valsartan to enhance patient outcomes and optimize healthcare resource utilization. However, further research is needed, particularly in elderly and renal-impaired populations, to provide a more comprehensive understanding of these medications' long-term efficacy and safety profiles.
